# Supplementary material for: Use of assistive technology to assess distal motor function in subjects with neuromuscular disease
Source: PLOS Digit Health. 2025 Jan 13;4(1):e0000534. doi: 10.1371/journal.pdig.0000534 (PMC11729976; doi:10.1371/journal.pdig.0000534)
Supplement: S1 File — (PDF) [file pdig.0000534.s001.pdf]

Supporting Information 1: Mathematical representation and processing carried out by TabMe2 software

Item18

For the mathematical processing of the drawings for item 18, the following parameters were used: zones, minimum angle for making the circle, distance between adjacent traces and speed threshold (minimum).

The CD image is divided into three zones described in Fig A (A1, A2 and A3). The subject starts with a finger positioned in zone A1. To obtain a score of 1, the complete circular drawing must lie entirely within area A2. Within zone A3, a complete circular drawing without compensation will be scored 3, or 2 if compensation is observed.

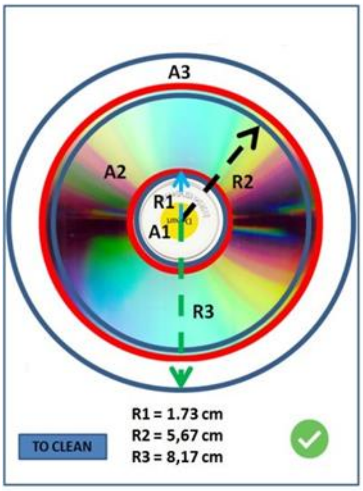

<u>Fig 1:</u> Definition of 3 zones on the screen of the digital interface of item 18

In the event of motor difficulty, the subject may have to change fingers, which limits the score to 2 and can be represented by 2 consecutive traces. The model considers that 2 consecutive traces are joined only if they are no more than 1.5 cm apart (which corresponds to 150 pixels on the tablet screen). The support of the wrist or palm or other fingers is also recorded, as they limit the scoring to 2. While the subject is drawing, the system time is recorded continuously with each point touched. By calculating the distance between adjacent points and the time difference between them, the average drawing speed is calculated (moving average of 10 samples). To avoid being recorded as a compensation, the average speed must not be less than 0.5 cm/s. To avoid false negatives at the start of the drawing because of the starting position, finger placed in the centre of the CD, the check is only triggered when the threshold of 1 cm/s is reached.

Item 19

For the mathematical processing of the drawings, the following parameters were used: the zones and the speed threshold (minimum). The image of the rectangle is divided into 3 zones described in Fig 2.

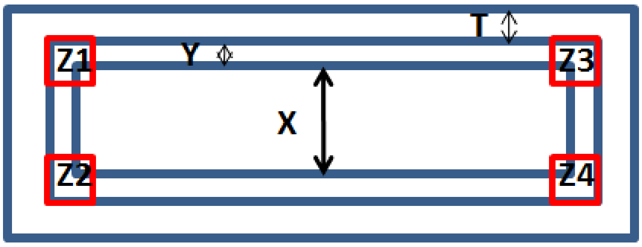

Fig 2: Definition of 3 zones on the screen of the digital interface of item 19: X= 1 cm, Y= 0.1 cm, T = 0.5 cm. Definition of 4 corner zones Z.

The drawing must be done in the X+Y zone, the total dimensions of which are 1.2 cm x 4.2 cm. Any drawing made with the pen within zone X guarantees a mark of 1. To be considered a complete loop, the vertical ends of the loop must be confined within zone Y. For a score of 2, at least one complete loop must be drawn within the X+Y zone. For a score of 3, at least 3 loops must be drawn, and the horizontal ends of the entire design must also be confined within 2 of the 4 corners (Z1 to Z4) and all loops must be complete loops. Continuous movement is also required to achieve a score of 3. Drawing speed is analysed in the same way as in point 18. A loss of pen contact during the drawing process is considered as compensation and limits the score to 2.

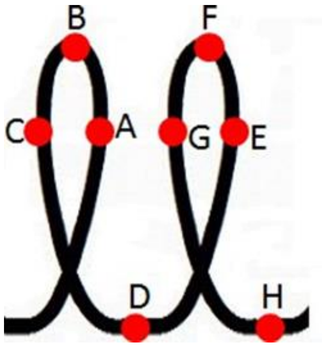

Fig 3: Inflection points for the definition of a loop of item 22. Horizontal inflection points: A, C, E and G. Vertical inflection points: B, D, F and H.

In order to distinguish the shape of a loop (from a triangle for example), the drawing is processed dynamically. As shown in Fig C, loops have 4 inflection points (2 vertical, 2 horizontal), and adjacent loops have corresponding horizontal inflection points which evolve over time by increasing their horizontal coordinate. In addition, each horizontal inflection point is followed by a vertical inflection point (and vice versa). As explained in the previous subsection, the vertical inflection points must be confined to the Y zone.

Fig 4A and B shows examples of scores 2 and 3 of item 19.

A

B

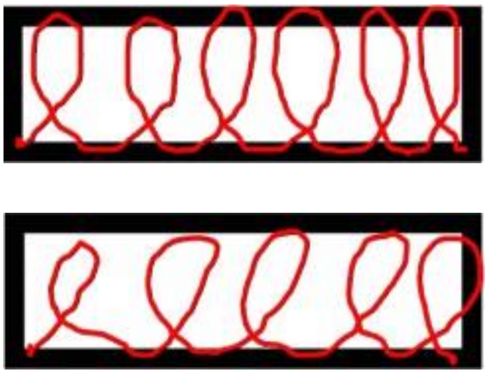

<u>Fig 4:</u> Illustration of a score 3 of item 19 (A) and illustration of a score 2 of item 19 (B); all the loops do not have vertical limits confined to the Y zone.

A minimum speed threshold has been set at 0.1 cm/s (corresponding to 10 pixels/s on the tablet screen) to define a pause in the loops, which limits the score to 2.

<u>Item 22</u>

Unlike the previous items, in which a score of 3 is obtained with a single trace drawing. The maximum score for item 22 was associated with the patient's ability to touch the inside of the figures in a diagram without sliding. To detect slipping, the boundaries of each of the 9 boxes on the diagram had to be defined (Fig 5).

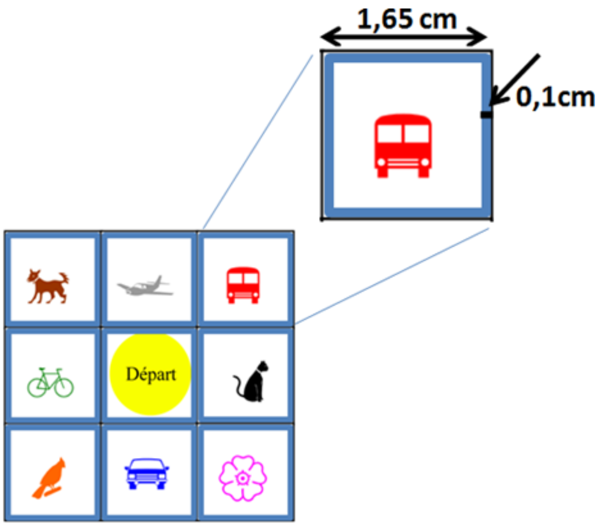

<u>Fig 5:</u> Touch zone limits for item 22

The subject starts with a finger positioned in the centre of the diagram on the word "start". For a score 3, the subject raises the finger and places it successively on the 8 drawings of the diagram without touching the lines. In this case, the contact of the finger on the tablet screen is characterised by a circular shape with a radius of 0.8 cm, centred on the first point touched on the screen. Detection of finger contact with the blue line requires a boundary spacing of 0.1 cm from the black line (Fig 5). If the

finger touches the lines, the score is limited to 2. The score 1 is for a subject who cannot raise the finger to place it on a drawing, but can slide it on at least one drawing.
