# Supplementary material for: Use of assistive technology to assess distal motor function in subjects with neuromuscular disease
Source: PLOS Digit Health. 2025 Jan 13;4(1):e0000534. doi: 10.1371/journal.pdig.0000534 (PMC11729976; doi:10.1371/journal.pdig.0000534)
Supplement: S1 Table — (PDF) [file pdig.0000534.s002.pdf]

Supporting Information 2: Illustrations of A-score for disagreements between scores of the therapist's score on the tablet (T-score) and the automatic score (A-score) from the TabMe2 software

A (Item 18)

Problem encountered	n	Score A given	Item 18
Problem with instructions	6	0	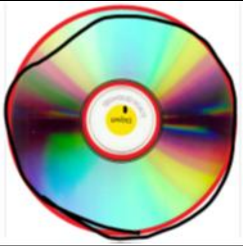
Problem with the use of the digital interface	6	0	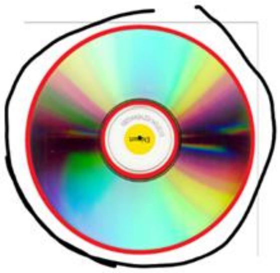
Calibration issues	4	0	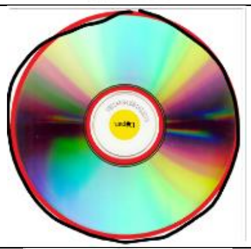
Difference in analysis of speed of item completion	2	2	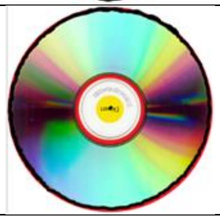
Postural compensation not seen	3	3	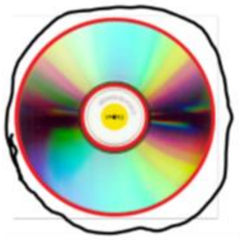
No reason found	1	0	

B (item 19)

Problem encountered	n	Score A given	Item 19
Calibration issues	2<br>1	2<br>1	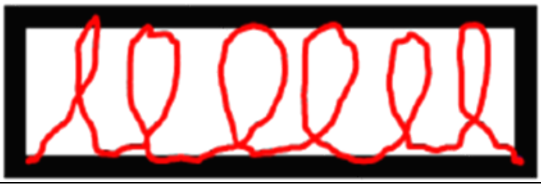

C (item 22)

Problem encountered	n	Score A given	Item 22
Calibration issues	3	2	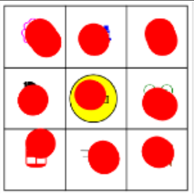
